# Does provision of antenatal care in Southern Asia improve neonatal survival? A systematic review and meta-analysis

**DOI:** 10.1016/j.xagr.2022.100128

**Published:** 2022-11-07

**Authors:** Millie A. O'Dair, Andrew Demetri, Gemma L. Clayton, Deborah Caldwell, Katie Barnard, Christy Burden, Abigail Fraser, Abi Merriel

**Affiliations:** a Academic Women's Health Unit, Bristol Medical School, University of Bristol, Bristol, United Kingdom (O'Dair, Demetri, Barnard, Burden, Merriel); b Medical Research Council Integrative Epidemiology Unit, University of Bristol, Bristol, United Kingdom (Clayton); c Population Health Sciences, Bristol Medical School, University of Bristol, Bristol, United Kingdom (Caldwell, Fraser); d North Bristol National Health Service Trust, Bristol, United Kingdom (Burden); e National Institute for Health and Care Research Biomedical Research Centre, Bristol Medical School, University of Bristol, Bristol, United Kingdom (Burden, Fraser, Merriel); f Centre for Women's Health Research, Institute of Life Course and Medical Sciences, Faculty of Health & Life Sciences, University of Liverpool, Liverpool, United Kingdom (Merriel)

**Keywords:** antenatal care, maternal health services, neonatal death, neonatal mortality, prenatal care, Southern Asia

## Abstract

**BACKGROUND:**

Southern Asia has one of the highest burdens of neonatal mortality worldwide (26/1000 live births). Ensuring that women receive antenatal care from a skilled provider may play an important role in reducing this burden.

**OBJECTIVE:**

This study aimed to determine whether antenatal care received from a skilled provider could reduce neonatal mortality in Southern Asia by systematically reviewing existing evidence.

**STUDY DESIGN:**

Seven databases were searched (MEDLINE, Embase, Cochrane Library, CINAHL, PubMed, PsycINFO, and International Bibliography of the Social Sciences [IBSS]). The key words included: “neonatal mortality,” “antenatal care,” and “Southern Asia.” Nonrandomized comparative studies conducted in Southern Asia reporting on neonatal mortality in women who received antenatal care compared with those who did not were included. Two authors carried out the screening and data extraction. The Risk of Bias Assessment tool for Non-randomized Studies (RoBANS) was used to assess quality of studies. Results were reported using a random-effects model based on odds ratios with 95% confidence intervals.

**RESULTS:**

Four studies were included in a meta-analysis of adjusted results. The pooled odds ratio was 0.46 (95% confidence interval, 0.24 to 0.86) for neonatal deaths among women having at least 1 antenatal care visit during pregnancy compared with women having none. In the final meta-analysis, 16 studies could not be included because of lack of adjustment for confounders, highlighting the need for further higher-quality studies to evaluate the true impact.

**CONCLUSION:**

This review suggests that in Southern Asia, neonates born to women who received antenatal care have a lower risk of death in the neonatal period compared with neonates born to women who did not receive antenatal care. This should encourage health policy to strengthen antenatal care programs in Southern Asia.

**AJOG Global Reports at a Glance**

**Why was this study conducted?**

Increasing the uptake of antenatal care could be an effective way to reduce high neonatal mortality rates in Southern Asia. However, previous research assessing interventions has often been inconsistent.

**Key findings**

Neonatal death rates were lower in women who received any antenatal care than in those who did not receive any.

**What does this add to what is known?**

Increasing antenatal care uptake in Southern Asia could reduce neonatal deaths.

## Introduction

Every year there are 2.5 million neonatal deaths (defined as “death of a liveborn during the first 28 days of life”) globally.^1^ Neonatal deaths make up 45% of all deaths in children aged <5 years.^2^ Between 2000 and 2016, global neonatal mortality fell by 39%, whereas mortality among children aged <5 years fell more quickly by 47%.^3^^,^^4^ Despite this, most focus is placed on interventions outside the neonatal period.^4^^,^^5^

Vast disparities in neonatal mortality rates (NMRs)^1^ exist between high- and low-income countries, with NMRs <3 in most high-income countries, as opposed to an average of 27 in low-income countries.^6^ Southern Asia has one of the highest burdens of neonatal deaths worldwide, which make up 59% of deaths among children aged <5 years in the region.^3^^,^^7^ It has been slow to reduce its NMR of 26 per 1000.^1^^,^^2^

Disparities also exist in causes of neonatal deaths. For example, 4 infectious diseases (sepsis, pneumonia, tetanus, and diarrhea) make up 7% of neonatal deaths in high-income countries, as opposed to 23% of deaths in Southern Asia.^3^ This suggests that there are interventions available that high-income countries are using to prevent these deaths, but low-income countries are not benefiting from them. It is estimated that 71% of neonatal deaths could be prevented each year with increasing coverage of existing interventions in the antenatal, intrapartum, and postnatal periods.^8^

Antenatal care (ANC), defined by the World Health Organization (WHO) as “care provided by skilled healthcare professionals to pregnant women and adolescent girls to ensure the best health conditions for both mother and baby,” is an opportunity to optimize health before birth and identify pregnancy risk.^5^^,^^9^^,^^10^ There is high-quality evidence that specific antenatal interventions (such as iron and folate supplementation, tetanus toxoid immunizations, and screening for preeclampsia) improve neonatal outcomes.^9^^,^^11^

It is important to emphasize the difference that ANC could make in reducing neonatal mortality in Southern Asia. This is especially important given that until recently, most pregnancy care in Southern Asia was carried out by traditional birth attendants, despite skilled birth attendants having become a central tenet of improving pregnancy outcomes.^12^ Few studies have focused on ANC delivery more generally, rather than on specific interventions.^9^^,^^13^^,^^14^

This systematic review aimed to assemble evidence that receiving ANC with a skilled provider can reduce neonatal mortality in Southern Asia when compared with receiving no ANC or receiving ANC with a nonskilled provider. Such evidence would help inform policy makers and incentivize more focus on and funding for ANC.

## Materials and Methods

### Search strategy

Seven electronic databases (MEDLINE, Embase, PubMed, CINAHL, International Bibliography of the Social Sciences [IBSS], PsycINFO, and Cochrane Library) were systematically searched for eligible studies from inception to June 2022 and restricted to English language. Search terms used included: “neonatal mortality,” “neonatal death,” “antenatal care,” “prenatal care,” and “Southern Asia.” These were searched as both subject headings and free text. Details of the full search strategy can be found in Supplementary File 1.

### Study selection

Results were imported into Covidence systematic review software (Veritas Health Innovation, Melbourne, Australia)^15^ and deduplicated before title and abstract screening. Irrelevant papers were excluded, and full texts of remaining studies retrieved and screened for eligibility. All screening was carried out independently by 2 reviewers, and any disagreements were resolved through discussion between them with arbitration from a third author if necessary.

### Eligibility criteria

Studies were included if they were carried out in 1 of the 8 countries of Southern Asia (India, Bangladesh, Nepal, Afghanistan, Pakistan, Sri Lanka, Bhutan, Maldives), and if they reported on risk of neonatal mortality and on participation in ANC, whether this was based in a health facility or community setting. Studies that implied that the ANC was received in a formal healthcare setting rather than from a traditional birth attendant were included. Only nonrandomized studies were included because it would be unethical to have a randomized control trial where ANC was withheld from a control group.

Studies were excluded if they: were not in English; included high-risk pregnancies only; did not separate neonatal deaths from stillbirths; or only focused on the number of ANC visits received and had no comparator of no ANC.

### Data extraction

Data were extracted independently by 2 reviewers into a standardized data-extraction form. Data were compared, and differences resolved through discussion or with arbitration from a third reviewer if necessary. Data extracted included: country, study setting and design, sample size, and outcome and exposure definitions. The primary outcome was neonatal mortality, defined as death of a live-born infant during the first 28 days of life. If studies reported data on number of ANC visits received, this was also recorded. The actual counts and unadjusted effect estimates were recorded (eg, odds/risk/hazard ratios with 95% confidence intervals [CIs]). Odds ratios (ORs) were calculated from raw data if not given in the studies. Corresponding authors were contacted for additional information when studies did not provide enough data to calculate estimates. Adjusted effect estimates were also recorded, and the variables were included in multivariable models.

### Quality assessment

The Risk of Bias Assessment tool for Non-randomized Studies (RoBANS)^16^ was used to assess the quality of included studies. Six domains were assessed: selection bias, confounding bias, performance bias, detection bias, attrition bias, and reporting bias. Each was allocated as “low risk,” “high risk,” or “unclear.”

### Data synthesis

A Mantel–Haenszel random-effects model was used to perform the meta-analysis. Heterogeneity was assessed using the I^2^ statistic.^17^ Studies that did not provide enough data to calculate effect estimates and whose authors did not respond were excluded from the meta-analysis. We performed planned subgroup analyses of number of ANC visits (for 1–3 visits and ≥4 visits) by country.

### Protocol and guidance

This systematic review was conducted according to the Preferred Reporting Items for Systematic Reviews and Meta-Analyses (PRISMA) guidelines (Supplementary File 2).^18^ The protocol of this review was registered with the International Prospective Register of Systematic Reviews (PROSPERO; registration number CRD42020177148).

## Results

### Search results

After deduplication, 5099 studies remained for title and abstract screening. Full-text review was carried out for 152 papers, and 21 records of 20 studies met the inclusion criteria. Main reasons for exclusion were not having “no ANC” as a comparator, wrong outcome (eg, perinatal mortality), or ANC being combined with other interventions and not assessed as a comparator alone (shown in PRISMA flow diagram in Supplementary File 3).

### Study characteristics

Unadjusted data were provided on 292,888 mothers; 87,308 women received no ANC and had 2485 (2.85%) neonatal deaths. There were 3359 neonatal (1.87%) deaths among the 184,261 mothers who received ANC. However, adjusted analyses were presented only in 4 studies including 49,458 mothers; these studies constitute our primary analysis.^19^^,^^27^^,^^29^^,^^39^ There were 14,469 mothers who received no ANC, of whom 284 had neonatal deaths (1.96%), and 30,276 mothers received ANC, of whom 373 had neonatal deaths (1.23%).

Studies from 5 of the 8 Southern Asian countries were included in the review. Nine studies were based in Bangladesh,^19^, ^20^, ^21^, ^22^, ^23^, ^24^, ^25^, ^26^, ^27^ 5 in India,^28^, ^29^, ^30^, ^31^, ^32^ 5 in Nepal,^33^, ^34^, ^35^, ^36^, ^37^ 1 in Pakistan,^38^ and 1 in Afghanistan.^39^ Fifteen of 20 studies were cross-sectional,^19^^,^^20^^,^^23^, ^24^, ^25^, ^26^, ^27^, ^28^^,^^30^^,^^32^, ^33^, ^34^, ^35^^,^^37^, ^38^, ^39^ with data for both exposure and outcome obtained from household questionnaires as part of national demographic and health surveys. Three studies were case–control,^21^^,^^31^^,^^36^ 2 were prospective cohort studies,^22^^,^^29^ and 1 was a retrospective cohort study.^28^ These also used household questionnaires, and thus both exposure and outcomes were self-reported measures. Included studies were published between 1987 and 2021. A summary of included study characteristics is presented in Supplementary File 4.

### Risk of bias

Of the 20 studies, 19 had at least 1 area of high or unclear risk of bias. Six studies had 3 areas of high risk,^24^^,^^25^^,^^30^^,^^34^^,^^35^^,^^37^ and another 6 studies had 2 areas of high risk.^20^^,^^23^^,^^32^^,^^33^^,^^36^^,^^39^ The results of the risk of bias (RoBANS) assessment can be found in Table 1.

**Table 1:** Risk of Bias Assessment tool for Non-randomized Studies (RoBANS)

Author/year	Selection of participants	Confounding	Measurement of exposure	Blinding of outcome assessments	Incomplete outcome data	Selective outcome reporting
Abir T, 2017^19^	Low	Low	High	Low	Low	Low
Akter T, 2017^20^	High	Low	High	Low	Low	Low
Bapat U, 2012^29^	Low	Low	Low	Low	Low	Low
Ghimire PR, 2019^33^	Low	High	High	Low	Low	Low
Ghosh R, 2010^30^	High	High	High	Low	Low	Low
Islam M, 2021^27^	Low	Unclear	Unclear	Low	Low	Low
Kibria GMA, 2018^39^	High	Low	High	Low	Low	Low
Mavalankar DV, 1991^31^	Low	High	Low	Low	Low	Low
Mercer A, 2006^21^	Low	High	Low	Low	Low	Low
Neupane S, 2014^34^, ^35^	High	High	High	Low	Low	Low
Nisar YB, 2014^38^	High	High	High	Low	Low	Low
Owais A, 2013^22^	Low	High	Low	Low	Low	Low
Rahman A, 2009^23^	High	Low	High	Low	Low	Low
Rahman A, 2010^24^	High	High	High	Low	Low	Low
Roy S, 2010^25^	High	High	High	Low	Low	Low
Shah R, 2015^36^	Low	High	High	Low	Low	Low
Shakya K, 2001^37^	High	High	High	Low	Low	Low
Singh A, 2008^32^	High	High	Low	Low	Low	Low
Torre M N, 2021^28^	Low	High	Low	Low	Low	Low
Uddin J, 2008^28^	Low	Low	High	Low	Low	Low

Summary of the risk of bias assessment for each domain across studies.

*O'Dair. Does provision of antenatal care improve neonatal survival? Am J Obstet Gynecol Glob Rep 2022.*

Reporting bias was found to be low in all studies because they all reported their predefined outcomes in the results. All studies had low risk of attrition bias because they all had <10% missing data. Detection bias was also low in all studies because although blinding was performed by few studies, it was unlikely to affect the outcomes.

Ten studies had high risk of selection bias primarily owing to the inclusion of only “ever married” women. The remaining 10 were of low risk for selection bias because they included all women who had live-born infants in the time frame and area of the study.^19^^,^^21^^,^^22^^,^^26^, ^27^, ^28^, ^29^^,^^31^^,^^33^^,^^36^

Only 5 studies adjusted for appropriate confounders such as socioeconomic status, place of residence, maternal age, and maternal education.^19^^,^^20^^,^^23^^,^^29^^,^^39^ The other 15 studies did not consider potential confounding variables, and were therefore of high risk.

Fourteen studies relied on recall for measurement of exposure. Only 6 used medical records or interviews during and immediately after pregnancy, and therefore had low risk of performance bias.^21^^,^^22^^,^^28^^,^^29^^,^^31^^,^^32^

### Data synthesis

Only 4 studies provided adjusted ORs.^19^^,^^27^^,^^29^^,^^39^ Confounders adjusted for in 3 included studies were place of residence, socioeconomic status, maternal education, and the sex of the infant. Mother's age, parity, pregnancy interval, and employment of parents were adjusted for in 2 of the studies. One study did not explain which confounders it adjusted for.^27^

**Figure 1:**
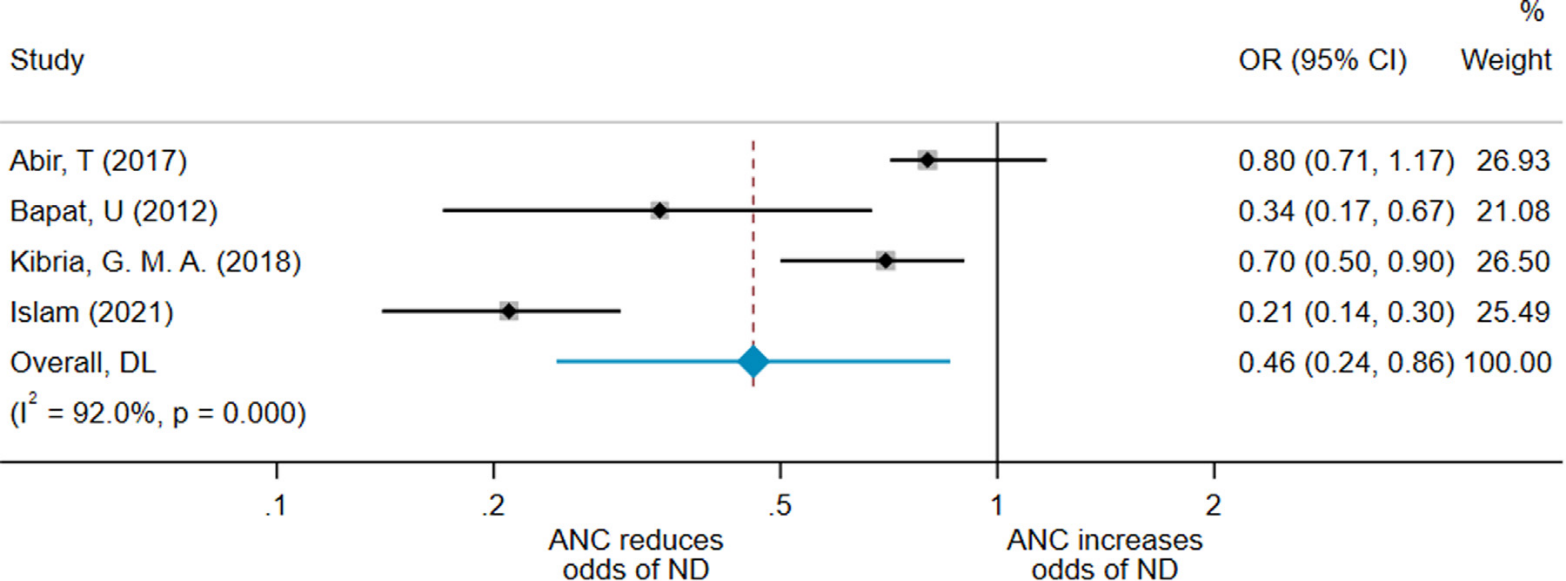
Forest plot for 4 adjusted meta-analysis studies A forest plot showing the result of the meta-analysis including the 3 studies that adjusted for confounders.^19^^,^^29^^,^^39^ *ANC*, antenatal care; *CI*, confidence interval; *DL*, Random effects (DerSimonian-Laird - DL) meta-analysis; *ND*, neonatal death; *OR*, odds ratio. *O'Dair. Does provision of antenatal care improve neonatal survival? Am J Obstet Gynecol Glob Rep 2022.*

**Figure 2:**
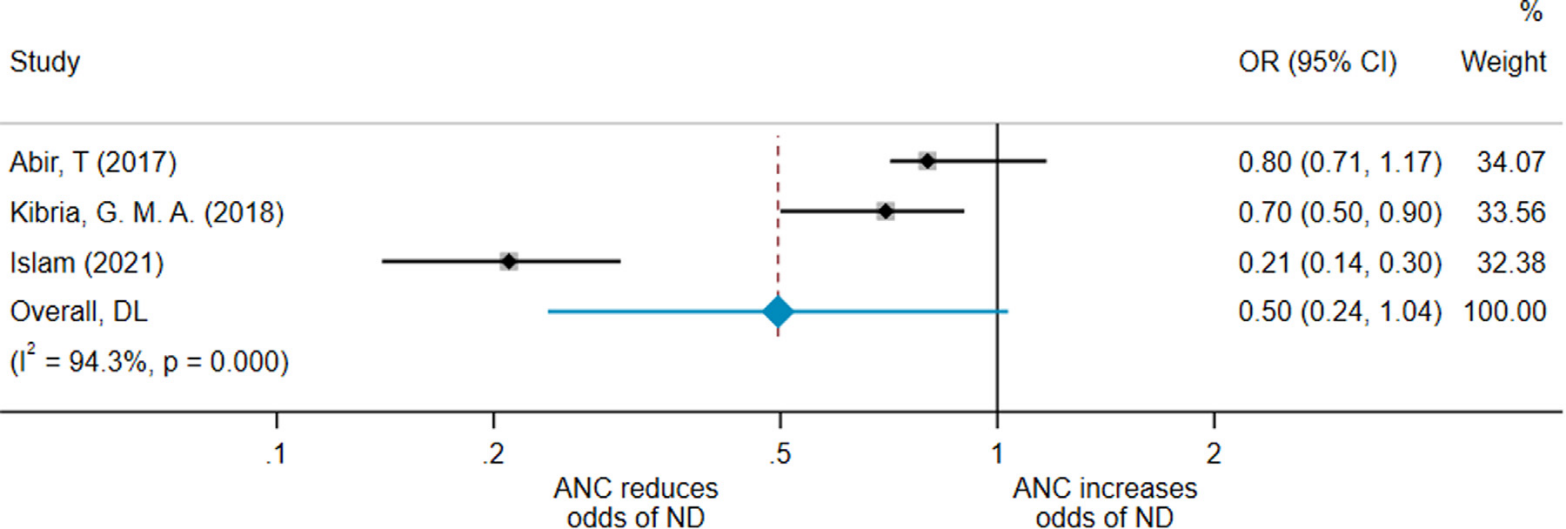
Forest plot for 3 adjusted cross-sectional meta-analysis studies A forest plot showing the result of the meta-analysis of the 2 cross-sectional studies that adjusted for confounders.^19^^,^^39^ *ANC*, antenatal care; *CI*, confidence interval; *DL*, Random effects (DerSimonian-Laird - DL) meta-analysis; *ND*, neonatal death; *OR*, odds ratio. *O'Dair. Does provision of antenatal care improve neonatal survival? Am J Obstet Gynecol Glob Rep 2022.*

As shown in Figure 1, the pooled effect from this adjusted meta-analysis by random-effects model gave an OR for neonatal death in the ANC group of 0.46 (95% CI, 0.24–0.86) compared with the no-ANC group. The *I*^2^=92.0%, indicating considerable heterogeneity across studies. We restricted analysis to studies of the same design to determine whether the heterogeneity was reduced. The pooled OR of the 3 cross-sectional studies^19^^,^^27^^,^^39^ was 0.50 (95% CI, 0.24–1.04; *I*^2^=94.3%) (Figure 2).

**Figure 3:**
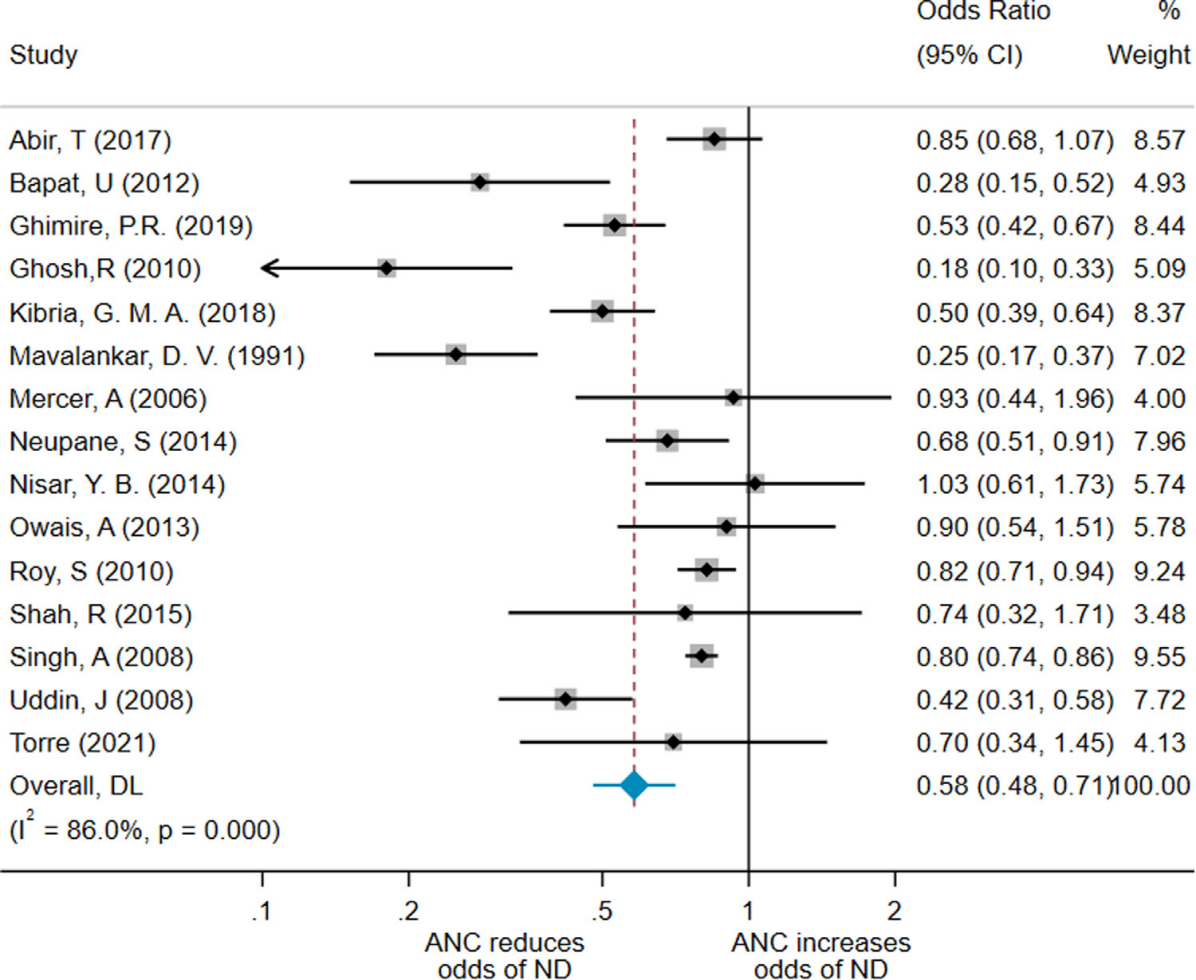
Forest plot for 15 nonadjusted meta-analysis studies A forest plot showing the result of the meta-analysis of 14 studies using unadjusted results. *ANC*, antenatal care; *CI*, confidence interval; *DL*, Random effects (DerSimonian-Laird - DL) meta-analysis; *ND*, neonatal death. *O'Dair. Does provision of antenatal care improve neonatal survival? Am J Obstet Gynecol Glob Rep 2022.*

The pooled effect from the meta-analysis of unadjusted results from 3 of these studies gave an OR of 0.53 (95% CI, 0.31–0.90; *I*^2^=87.4%), as shown in Supplementary File 5. One could not be included because of lack of providing raw data.^27^ This shows that the effect of the unadjusted ORs overestimated the ANC effect, as expected. The pooled effect of the 3 cross-sectional studies gave an OR of 0.66 (95% CI, 0.39–1.10; *I*^2^=88.2%) (Supplementary File 6). For completion, we performed a meta-analysis of unadjusted ORs for the 15 studies that provided raw data (Figure 3). The pooled effect from this meta-analysis was an OR of 0.58 (95% CI, 0.48–0.71; *I*^2^=86%). It is promising that these are in the same direction of the results of adjusted studies.

We planned, a priori, to perform a subgroup analysis by country; however, this was not possible using adjusted ORs. For completion, we undertook subgroup analysis by country using unadjusted, crude estimates, and these are presented in Supplementary File 7.

This analysis showed that the OR for neonatal death was 0.73 for Bangladesh (95% CI, 0.56– 0.96; *I*^2^=75.3%), 0.37 for India (95% CI, 0.18–0.77; *I*^2^=95.4%), and 0.59 for Nepal (95% CI, 0.49–0.71; *I*^2^=0.9%). There was only 1 study included from Afghanistan and Pakistan.

Four studies provided insufficient data to be incorporated into any meta-analysis.^20^^,^^23^^,^^24^^,^^37^ However, all 4 reported lower neonatal mortality in women receiving ANC compared with women who did not. The individual results of these studies are summarized in Table 2.

**Table 2:** Summary of results of studies that could not be included in the meta-analysis

Study	Result for women who received ANC compared with those who received none
Akter et al (2017)^20^	Hazard ratio of 0.77 (95% CI, 0.49–1.22)
Rahman et al (2009)^23^	Hazard ratio of 0.657 (no CI given)
Rahman et al (2010)^24^	Decrease in NMR of 10/1000 live births
Shakya et al (2001)^37^	Crude odds ratio of 0.68 (no CI)

ANC, antenatal care; CI, confidence interval; NMR, neonatal mortality rate.

*O'Dair. Does provision of antenatal care improve neonatal survival? Am J Obstet Gynecol Glob Rep 2022.*

### Number of antenatal care visits

**Figure 4:**
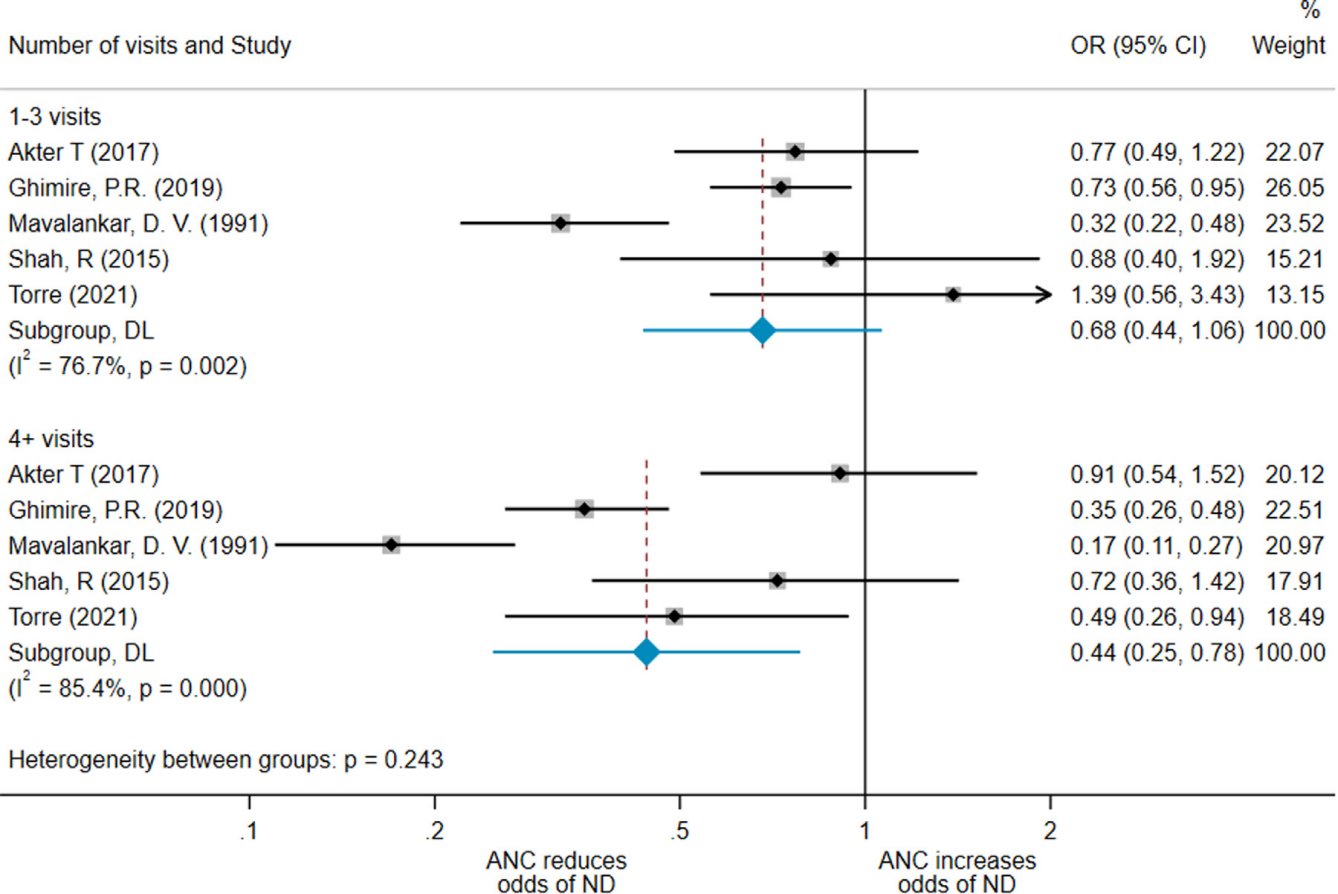
Forest plot for subanalysis by number of visits received A forest plot showing the result of the meta-analysis performed as a subanalysis of number of antenatal care visits received, using unadjusted results. *ANC*, antenatal care; *CI*, confidence interval; *DL*, XXX; *ND*, neonatal death; *OR*, odds ratio. *O'Dair. Does provision of antenatal care improve neonatal survival? Am J Obstet Gynecol Glob Rep 2022.*

An analysis examining neonatal mortality by number of ANC visits was planned a priori but could not be conducted using results adjusted for confounders. We therefore used unadjusted results from 5 studies^20^^,^^28^^,^^31^^,^^33^^,^^36^ and obtained an OR of 0.68 (95% CI, 0.44–1.06; *I*^2^=76.7%) for women receiving 1 to 3 visits and 0.44 (95% CI, 0.25–0.78; *I*^2^=85.4%) for women receiving ≥4 visits (Figure 4).

Four other studies reported data on number of ANC visits that women received, but could not be included in meta-analysis because of differences in how data were reported. The individual results of these studies are described in Table 3. These results are more varied and modest than those of our meta-analysis, but do nevertheless suggest a positive association between number of ANC visits received and reduction in neonatal mortality.

**Table 3:** Summary of results of studies reporting on effect of number of ANC visits which could not be included in a meta-analysis

Study	Results
Shakya et al (2001)^37^	2–3 visits compared with no ANC: OR, 0.71 ≥4 visits: OR, 0.77
Rahman et al (2010)^24^	1–2 visits compared with no ANC: OR, 0.65 (95% CI, 0.47–0.79) ≥3 visits: OR, 0.46 (95% CI, 0.33–0.56)
Uddin et al (2008)^26^	Percentage decrease in neonatal death: 1–2 visits: 3.31% 3–4 visits: 1.58% ≥5 visits: 1.54%
Mercer et al (2006)^21^	1–2 visits compared with no ANC: OR, 1.05 (95% CI, 0.48–3.65) ≥3 visits: OR, 0.89 (95% CI, 0.42–1.87)

ANC, antenatal care; CI, confidence interval; OR, odds ratio.

*O'Dair. Does provision of antenatal care improve neonatal survival? Am J Obstet Gynecol Glob Rep 2022.*

## Comment

### Principal findings

Only 4 studies provided results accounting for potential confounders. Two of these studies had high risk of performance bias because of how exposure was measured,^19^^,^^39^ and one had high risk of selection bias.^39^ One study had an unclear risk of confounding and performance bias because it did not explain how ANC was measured or which confounders it adjusted for.^27^ Primary analysis of adjusted results showed that receiving any ANC was associated with a 54% reduction in neonatal mortality in Southern Asia. The other analyses of unadjusted results further supported this finding, but conclusions drawn from these are limited.

The subgroup analysis by country suggested differences in effect of ANC on neonatal deaths between the countries within Southern Asia. For example, it suggested that in Nepal, ANC could result in a 63% reduction in neonatal death as opposed to 27% in Bangladesh. The meta-analysis by number of visits suggested that receiving more ANC visits may lead to a greater reduction in neonatal mortality. However, we cannot rely on these conclusions because only unadjusted results were used, and many of the studies used in this subgroup analysis had multiple domains of high bias.

### Strengths and limitations

The major strength of this study is that it was a systematic review investigating the association between receiving any ANC by a skilled provider and neonatal mortality in Southern Asia. Previous studies on the effect of ANC on neonatal mortality have provided strong evidence for the benefit of individual antenatal interventions, but few have provided evidence for the benefits of ANC as a whole.^9^^,^^11^^,^^13^

The greatest limitation was because of studies not accounting for potential confounders in reporting results. It is well-established that socioeconomic factors such as income, education, and place of residence/region are associated with access to health care, healthcare–seeking behaviors, and pregnancy outcomes/mortality.^4^^,^^9^^,^^14^ Therefore, not accounting for these factors when nterpreting results could cause an overestimation of the effect of ANC. Our results are promising, but there is need for further studies that account for confounders to evaluate the true impact.

In the adjusted meta-analysis, there was a high level of heterogeneity. When considering only studies of cross-sectional design, there was minimal heterogeneity and the effect was in the same direction, although slightly attenuated compared with the overall analysis. However, there could have been many other factors contributing to the heterogeneity. The exposure of receiving ANC was poorly defined in most studies. Although studies focusing on ANC provided by nonmedical trainees (eg, traditional attendants) were excluded, only 8 studies specifically stated that the ANC was received from a skilled provider.^19^, ^20^, ^21^^,^^26^^,^^34^^,^^35^^,^^38^^,^^39^ However, the remaining studies implied that ANC was formal rather than provided by, for example, a traditional birth attendant. The other 12 studies did not specify who provided the ANC; therefore, it is possible that there may have been differences in ANC providers across the studies, which could explain the substantial heterogeneity among studies. Given that NMRs and ANC coverage vary significantly among the countries in Southern Asia, differences in study setting could have also contributed to this heterogeneity.^1^^,^^40^

Meta-analysis by number of visits is useful because it can contribute to the argument about the importance of women receiving the recommended number of ANC visits, as per WHO guidelines.^10^ However, we were unable to analyze this as robustly as we would have liked because of the small number of studies reporting this information and the variations in how they reported the number of visits.

### Result

This review suggests that receiving any level of ANC can contribute to a significant reduction in neonatal mortality in Southern Asia. Our result is in line with previous evidence, given that studies looking at risk factors of neonatal deaths have suggested that poor ANC is associated with neonatal mortality.^41^^,^^42^ It is promising that a review focusing on sub-Saharan Africa came to a similar conclusion, showing that use of ANC can reduce risk of neonatal mortality by 39%.^43^ Given that we focused on neonatal deaths, and therefore studies that included stillbirths were excluded, it is possible that the effect of ANC was underestimated in all the studies because the effects of ANC on even achieving a live birth were disregarded. We have shown that an important method of reducing NMRs in Southern Asia could be to prioritize ANC.

The proportion of women having only 1 ANC visit in Southern Asia is 79%—the second lowest globally following West and Central Africa.^40^ Only 49% of women in Southern Asia and as few as 17.8% of women in Afghanistan have 4 visits, which is much lower than the 80% coverage target for providing 4 antenatal visits.^40^ The high NMR in Southern Asian countries could reflect low uptake of ANC.

The group of women who received no ANC at all likely represent a unique group, who may face difficulty accessing any care.^12^ Research into barriers that women face with regard to attending ANC in Southern Asia provides important insight on why ANC coverage is so limited. A major theme among studies was the finding that many women viewed pregnancy as a normal, healthy condition and thus saw no reason to seek health care.^12^^,^^44^ Spiritual and religious beliefs also often play a part in women considering traditional methods over biomedicine. Many of the countries in Southern Asia have extreme poverty, and thus costs of ANC, both direct and indirect, can act as a barrier for many women.^12^ It was also found that often it is not the women who make decisions about their pregnancy. Close family members, such as the husband, mother, and mother-in-law, often make important decisions such as whether ANC is necessary.^12^^,^^45^ Greater focus on barriers faced by women regarding access to ANC is needed, with emphasis placed on improving accessibility and ensuring that the women and their families understand the benefits and importance of ANC.

### Research implications

Our results are promising and indicate that ANC does improve neonatal mortality. However, because of the poor quality (ie, primarily the lack of adjustment for confounders) of many of the studies included in this review, many could not be included in the final meta-analysis. Thus, higher-quality research about the implementation of ANC is required to ensure its effectiveness and increase its uptake in Southern Asia. Further research into the individual countries within Southern Asia could also be beneficial in ensuring that ANC is delivered uniformly and equally across Southern Asia.

Our evidence supports previous studies investigating the link between number of ANC visits and neonatal mortality, which provided promising evidence that increasing the frequency of visits can reduce the number of neonatal deaths.^46^, ^47^, ^48^ WHO guidelines now recommend that women have 8 ANC visits, which was updated from 4 because more recent evidence suggested that 8 visits were associated with fewer perinatal deaths.^10^^,^^13^ However, higher-quality research is also still needed to establish the correlation between the specific number of ANC visits and reduction in neonatal mortality. This evidence would help to prioritize translating international guidelines into local practice and ensuring that women receive the optimal level of ANC. However, given that we have concluded that attending any ANC results in better outcomes, one of the most important areas for research/policy change may involve enabling attendance of ANC for women who do not engage with services.

### Clinical implications

Our evidence, alongside the low rates of ANC uptake in Southern Asia, should help persuade health policy makers to invest more efforts into increasing the uptake of ANC, given that there is such considerable opportunity for improvement. This review further suggests that having at least 4 ANC visits is more effective at reducing neonatal mortality compared with having <4. Southern Asian countries rarely achieve 4 ANC visits,^40^ and aiming for 8 would therefore require significant investment. It may be useful to consider policy goals aiming to first achieve 4 visits, with a medium-term aim to increase to 8.

This study focused on whether ANC can be used to improve neonatal mortality. However, we know that ANC can also provide multiple other health benefits, as shown extensively in previous research,^5^^,^^9^, ^10^, ^11^ to both neonate and mother, which would be enhanced if policy makers successfully implemented strategies to ensure that all women receive adequate ANC.

## Conclusion

Ensuring that all women receive ANC during pregnancy could have an impact on preventing newborn death in Southern Asia. The uncertainty of the evidence regarding the high burden of neonatal deaths suggests that focus should be placed on ensuring that future observational studies are of high quality. Health policy makers focusing on reproductive health in Southern Asia should be encouraged to strengthen ANC programs and increase the allocation of funding.

## Appendix Supplementary materials

Image, application 1

Image, application 2

Image, application 3

Image, application 4

Image, application 5

Image, application 6

Image, application 7

## Footnotes

Supplementary material associated with this article can be found in the online version at doi:10.1016/j.xagr.2022.100128.
