# Supplementary material for: Does provision of antenatal care in Southern Asia improve neonatal survival? A systematic review and meta-analysis
Source: AJOG Glob Rep. 2022 Nov 7;2(4):100128. doi: 10.1016/j.xagr.2022.100128 (PMC9720596; doi:10.1016/j.xagr.2022.100128)
Supplement: Supplementary file 5 [file mmc5.docx]

**Supplementary file 1: Search Strategy from MEDLINE via Ovid (1946-present)**

| **#** | **Searches** | **Results** |
| --- | --- | --- |
| 1 | India/ OR Bangladesh/ OR Pakistan/ OR Afghanistan/ OR Nepal/ OR Sri Lanka/ OR Bhutan/ OR Maldives/ | 143462 |
| 2 | (India OR Bangladesh OR Pakistan OR Afghanistan OR Nepal OR Sri Lanka OR Bhutan OR Maldives).tw | 137530 |
| 3 | South* Asia.tw | 13093 |
| 4 | 1 OR 2 OR 3 | 209572 |
| 5 | ((Neonat* OR Neo-nat* OR Newborn* OR Baby OR Babies OR Infant) ADJ3 (Death* OR died OR die OR dies OR demise OR mortalit* OR fatal*)).tw | 41637 |
| 6 | Infant death/ | 224 |
| 7 | Infant mortality/ | 28362 |
| 8 | 5 OR 6 OR 7 | 58320 |
| 9 | Exp Prenatal care/ | 27075 |
| 10 | Exp Maternal health services/ | 48395 |
| 11 | Midwifery/ | 18960 |
| 12 | ((Antenatal* OR Ante-natal* OR Antepartum OR Ante-partum OR (Prenatal* OR Pre-natal* OR Perinatal* OR Peri-natal) OR (Matern* OR Pregnan* OR midwife* OR obstetric*)) ADJ3 (Care OR clinic* OR outpatient* OR appointment* OR visit* OR service*)).tw. | 71165 |
| 13 | 9 OR 10 OR 11 OR 12 | 112840 |
| 14 | 4 AND 8 AND 13 | 866 |
