# Supplementary material for: Does provision of antenatal care in Southern Asia improve neonatal survival? A systematic review and meta-analysis
Source: AJOG Glob Rep. 2022 Nov 7;2(4):100128. doi: 10.1016/j.xagr.2022.100128 (PMC9720596; doi:10.1016/j.xagr.2022.100128)
Supplement: Supplementary file 7 [file mmc7.docx]

**Supplementary file 4: Included Study Characteristics**

| **First Author, year of publication** | **Country** | **Study setting and design** | **Study period** | **Sample size** | **Outcome definition (neonatal mortality)** | **Source of exposure and outcome measurements** | **Exposure definition (antenatal care)** |
| --- | --- | --- | --- | --- | --- | --- | --- |
| **Abir T, 2017 [19]** | Bangladesh | Cross – sectional population based national study | 2004-2011 | 16714 | Death within 0-28 days | National survey with max 5-year recall | ANC which includes offering maternal health education, physical examination and interventions such as IFA supplementation and TT vaccination |
| **Akter T, 2017 [20]** | Bangladesh | Cross – sectional population based national study | 2006- 2011 | 7314 | Death during first 28 days of life | Standardised questionnaires with 5-year recall | ANC which is according to WHO, UNICEF and Guidelines for Bangladesh |
| **Bapat U, 2012 [29]** | India | Prospective cohort study -Urban (slums) community-based | October 2005 - September 2007 | 8449 | Liveborn but dies within 28 complete days | Interviews of participants randomly selected from a maternal and child vital register | ANC included in ‘health service factors’ |
| **Ghimire PR, 2019 [33]** | Nepal | Cross – sectional population based national study | 2001-2016 | 16792 | Death between 0-30 days | National survey with max 5-year recall | ANC included in ‘health service factors’ |
| **Ghosh R, 2010 [30]** | India | Cross- sectional peri-urban community-based study | January 2008 - March 2008 | 892 | Death occurring during the first 28 completed days of life | Structured interviews | Implied ANC was with a trained practitioner, included blood pressure, blood tests, abdominal exam and ultrasounds |
| **Islam A, 2021 [27]** | Bangladesh | Cross – sectional population based national study | 2014 survey | 4840 | Death of a live-born infant in the first four weeks of life | National Survey | Implied ANC was formal |
| **Kibria GMA, 2018 [39]** | Afghanistan | Cross – sectional population based national study | June 2015 -February 2016 | 19582 | Early neonatal death: death of a live-born baby within the first week of life | National survey | Mother received an antenatal check up by a skilled health care personnel |
| **Mavalankar DV, 1991 [31]** | India | Case -control study (Hospital based) | July 1987 - June 1988 | 1625 | Early neonatal death: death occurring in hospital in the first week of life | Standardised survey | Implied ANC was formal |
| **Mercer A, 2006 [21]** | Bangladesh | Case control (Rural community-based) | May 2004 - November 2004 | 739 | Death in first 28 days postpartum | Structured interviews | A medical check-up with a qualified practitioner (paramedic or MBBS doctor) |
| **Neupane S, 2014 [34,35]** | Nepal | Cross – sectional population based national study | 2006 Survey | 4136 | Dying within the first month of life | National survey with max 3-year recall | ANC visit with a health professional and excluding visits from traditional attendants |
| **Nisar YB, 2014 [38]** | Pakistan | Cross – sectional population based national study | 2002-2006 | 5702 | Death of a live born in the first month of life (birth to 30 days) | Standardised survey with max 5-year recall | ANC with a health professional excluding untrained providers |
| **Owais A, 2013 [22]** | Bangladesh | Prospective cohort study - rural community-based | January 2011-December 2011 | 516 | Death within 28 days of live birth | Structured interviews | Antenatal and obstetric health services |
| **Rahman A, 2009 [23]** | Bangladesh | Cross – sectional population based national study | 2004 Survey | 3759 | Death within 28 days of live birth | Standardised survey with max 5-year recall | ANC included in ‘health service factors’ |
| **Rahman A, 2010 [24]** | Bangladesh | Cross – sectional population based national study | January 2004 - May 2004 | 6,981 | Death of live born infants during neonatal period (first four weeks of life) | Standardised survey with max 5-year recall | Implied formal ANC by grouping with delivery with a skilled attendant and other health care services |
| **Roy S, 2010 [25]** | Bangladesh | Cross – sectional population based national study | December 2012 -March 2013 | 7753 | Early neonatal death: Neonates who died before reaching age 7 days after birth | Standardised questionnaire | Not clearly defined beyond “received antenatal care” |
| **Shah R, 2015 [36]** | Nepal | Case- control -rural community-based | April 2012 - July 2012 | 198 | Death of a newborn baby during the first 28 days of their life | Medical records | ANC included in ‘health service factors’ |
| **Shakya K, 2001 [37]** | Nepal | Cross – sectional population based national study | 1996 Survey | 4351 | Death in the neonatal period | Standard questionnaires with max 3-year recall | ANC included in ‘health service factors’ |
| **Singh A, 2008 [32]** | India | Cross – sectional population based national study | 2007-2008 | 171,529 | Death occurred during the first 28 completed days of life | National survey with max 3-year recall | Implied formal ANC by grouping with delivery with a skilled attendant and other health care services |
| **Torre M N, 2021 [28]** | India | Retrospective cohort | April 2017-December 2018 | 8840 | Death within the first month of life | Data collected from hospital records | Implied ANC was formal |
| **Uddin J, 2008 [26]** | Bangladesh | Cross – sectional population based national study | 1999-2000 | 6686 | Deaths within 0-28 days of age | Standard questionnaires with max 5-year recall | Utilisation of maternal health service in pregnancy |
